# Comprehensive analysis of CCCH zinc finger family in poplar (*Populus trichocarpa*)

**DOI:** 10.1186/1471-2164-13-253

**Published:** 2012-06-18

**Authors:** Guohua Chai, Ruibo Hu, Dongyuan Zhang, Guang Qi, Ran Zuo, Yingping Cao, Peng Chen, Yingzhen Kong, Gongke Zhou

**Affiliations:** 1 Key Laboratory of Biofuels, Chinese Academy of Sciences, Shandong Provincial Key Laboratory of Energy Genetics, Qingdao Institute of BioEnergy and Bioprocess Technology, Chinese Academy of Sciences, Qingdao, 266101, PR China; 2 Complex Carbohydrate Research Center, University of Georgia, 315 Riverbend Road, Athens, GA, 30602, USA

## Abstract

**Background:**

CCCH zinc finger proteins contain a typical motif of three cysteines and one histidine residues and serve regulatory functions at all stages of mRNA metabolism. In plants, CCCH type zinc finger proteins comprise a large gene family represented by 68 members in *Arabidopsis* and 67 in rice. These CCCH proteins have been shown to play diverse roles in plant developmental processes and environmental responses. However, this family has not been studied in the model tree species *Populus* to date.

**Results:**

In the present study, a comprehensive analysis of the genes encoding CCCH zinc finger family in *Populus* was performed. Using a thorough annotation approach, a total of 91 full-length CCCH genes were identified in *Populus*, of which most contained more than one CCCH motif and a type of non-conventional C-X_11_-C-X_6_-C-X_3_-H motif was unique for *Populus*. All of the *Populus* CCCH genes were phylogeneticly clustered into 13 distinct subfamilies. In each subfamily, the gene structure and motif composition were relatively conserved. Chromosomal localization of these genes revealed that most of the CCCHs (81 of 90, 90 %) are physically distributed on the duplicated blocks. Thirty-four paralogous pairs were identified in *Populus*, of which 22 pairs (64.7 %) might be created by the whole genome segment duplication, whereas 4 pairs seem to be resulted from tandem duplications. In 91 CCCH proteins, we also identified 63 putative nucleon-cytoplasm shuttling proteins and 3 typical RNA-binding proteins. The expression profiles of all *Populus* CCCH genes have been digitally analyzed in six tissues across different developmental stages, and under various drought stress conditions. A variety of expression patterns of CCCH genes were observed during *Populus* development, of which 34 genes highly express in root and 22 genes show the highest level of transcript abundance in differentiating xylem. Quantitative real-time RT-PCR (RT-qPCR) was further performed to confirm the tissue-specific expression and responses to drought stress treatment of 12 selected *Populus* CCCH genes.

**Conclusions:**

This study provides the first systematic analysis of the *Populus* CCCH proteins. Comprehensive genomic analyses suggested that segmental duplications contribute significantly to the expansion of *Populus* CCCH gene family. Transcriptome profiling provides first insights into the functional divergences among members of *Populus* CCCH gene family. Particularly, some CCCH genes may be involved in wood development while others in drought tolerance regulation. Our results presented here may provide a starting point for the functional dissection of this family of potential RNA-binding proteins.

## Background

Zinc-finger transcription factors, as one of the largest transcription factor (TF) families in plants, are critical regulators for multiple biological processes, such as morphogenesis, signal transduction and environmental stress responses [1,2]. They are characterized by the presence of common zinc finger motifs in which cysteines and/or histidines coordinate with a few zinc atoms to form the local peptide structures that are essential for their specific functions [3]. Most plant zinc-finger transcription factors (e.g. RING-finger, LIM, WRKY and DOF) regulate the gene expression with the aid of DNA-binding or protein-binding proteins [4-7]. Recently, a new type of *Arabidopsis* zinc-finger proteins, which differs from the previously identified plant zinc-finger TFs by regulating gene expression via directly binding to mRNA, was named as CCCH gene family [8].

The CCCH family contains a typical C3H-type motif and members of this family had already been identified in organisms from yeast to human [8-10]. The first identified CCCH member is hTTP (human tritetraproline) that can bind to class II AU-rich element (ARE) in the 3'-untranslated region (3'-UTR) of tumor necrosis factor α (TNFα) mRNA, in most cases, to mediate TNFα mRNA degradation [11,12]. Lately, more evidences support that several TIS11 proteins including hTTP, TIS11b and TIS11d can in concert regulate target mRNA degradation in RNA processing by similar mechanism [13,14]. Other CCCH proteins include *C. elegant* protein PIE-1 and POS-1 that can both control germ cell fate by inhibition of transcription or activation of protein expression from maternal RNAs [15,16].

Compared to the largely well-characterized CCCHs in animals, only a small number of CCCH proteins have been functionally characterized in *Arabidopsis* and rice. These CCCH proteins have been implicated to participate in a wide range of plant developmental and adaptive processes, including seed germination [17], embryo development [18,19], floral morphogenesis [20], plant architecture determination [21], FRIGIDA-mediated winter-annual habit [22], and leaf senescence [23]. In particular, two CCCH genes, *AtC3H14* (At1g66810) and *AtC3H15* (At1g68200), have recently been shown to act as the master regulators for secondary cell wall biosynthesis in *Arabidopsis*[24,25], which also suggests that their homologues may be involved in *Arabidopsis* secondary cell wall formation as well. Recently, accumulating evidences indicate that a number of CCCH genes participate in plant abiotic stresses and defense responses [8,24-26]. For example, two closely related proteins in *Arabidopsis*, AtSZF1 (salt-inducible zinc finger 1) and AtSZF2, both act as negative regulators in plant salt tolerance [26]. *Arabidopsis ZFAR1* encodes a zinc-finger protein with ankyrin-repeat domains, with its loss-of-function mutants showing increased local susceptibility to *Botrytis* and sensitivity to seed germination in the presence of abscisic acid (ABA) [27]. GhZFP1, a nuclear protein from Cotton, interacts with GZIRD21A and GZIPR5, and enhances drought, salt, salicylic acid (SA) stress and fungal disease tolerance in transgenic plants [28]. Recently, Wang and coworkers revealed that 11 subfamily IX members of *Arabidopsis* CCCH proteins were involved in conferring plant tolerance to different stresses such as drought, salt, cold shock and ABA [8].

Because of the economic importance in pulp and bio-fuel production, the studies on the genus *Populus* have been the hotspots for many years [29]. The completion of *Populus trichocarpa* genome sequence in 2006 makes it as a model tree for other tree species [30]. Although *Populus* and *Arabidopsis* are relatively closely related in the eurosid clade of the eudicots, they have strongly contrasting life cycle and adaptations to environmental stresses [31,32]. Since the CCCH gene family has the potential of associating with RNA as well as the critical functions in wood development and stress response, it was of interest for us to characterize the CCCH genes in *Populus*.

In this study, we report the comprehensive genomic identification and phylogenetic analysis of 91 members of CCCH gene family in *Populus trichocarpa*, as well as their expression profiling in six different tissues and under drought stresses. These *Populus* CCCH proteins were categorized into 13 subfamilies and exhibited diverse expression patterns, suggesting their functional differentiations. It is noteworthy that a subset of CCCH genes showed the highest level of transcript abundance in root and differentiating xylem. Among them, 12 genes were selected for investigation of their expression patterns by RT-qPCR analysis. Our preliminary results may provide the insights to further investigate the roles of these candidate genes in *Populus* differentiating xylem development and drought stresses.

## Results and Discussion

### Identification of CCCH gene family in *Populus*

The CCCH domain genes, characterized by the presence of 1–6 copies of CCCH-type zinc finger motifs, were already systematically analyzed in *Arabidopsis*, rice, human and *Trypanosoma*[8,10,33]. In the current study, to gain insight into the size of the CCCH gene family in *Populus*,the CCCH domains were used to screen the *Populus* genome database (release 2.1, http://www.phytozome.net/poplar.php) (see methods). These domains used as queries cover both the conventional (C-X_7_-C-X_5_-CX_3_-H and C-X_8_-C-X_5_-C-X_3_-H) and the recently defined non-conventional (e.g. C-X_4_-C-X_5_-CX_3_-H and C-X_11_-C-X_5_-C-X_3_-H) CCCH motifs. Initially, a total of 106 non-redundant putative CCCH genes were obtained. SMART and Pfam analysis were performed to remove those putative pseudogenes and incorrect annotated genes, and then resulted in 91 members recognized by either SMART (Sm00356) or Pfam (PF00642). Subsequently, manual reannotation was performed to correct the putative CCCH sequences using online web server FGENESH (http://linux1.softberry.com/berry.phtml). In this endeavor, 12 protein sequences were corrected for further analysis. Finally, all 91 *Populus* CCCH genes were manually verified for the presence of CCCH motifs using InterProScan program (http://www.ebi.ac.uk/Tools /InterProScan/). In comparison to the CCCH gene family in PlnTFDB (http://plntfdb.bio.uni-potsdam.de/v3.0/) and DPTF (http://dptf.cbi.pku.edu.cn/) where 99 and 69 members of CCCH gene family were deposited for *Populus* respectively, our result was roughly in agreement with PlnTFDB. All 91 identified *Populus* CCCH genes in our study were named as from *PtC3H1* to *PtC3H91* following the nomenclature proposed by the previous study [34].

The encoded proteins varied from 96 to 2120 amino acids (aa) in length with an average of 579 aa. The details on other parameters of nucleic acid and protein sequences were provided in Table 1 and Additional file 1. The number of predicted non-redundant CCCH genes in *Populus* (91) is greater than that in other representative species: *Arabidopsis*, rice, mouse, human and *Trypanosoma brucei* containing 68, 67, 58, 55 and 48 predicted CCCH genes, respectively [8,10,35]. The number of CCCH genes in *Populus* is roughly 1.34 fold of that in *Arabidopsis*, which is in consistency with the ratio of 1.4~1.6 putative *Populus* homologues to each *Arabidopsis* gene [30]. Similar to other transcription factor gene families [34,36], the presence of more CCCH genes in *Populus* further confirmed that the expansion of genome is common during *Populus* evolutionary process. This expansion appears to be arisen from multiple gene duplication events, including a whole-genome duplication event in the *Populus* lineage followed by multiple segmental and tandem duplication events [30].

**Table 1:** List of 91 CCCH genes identified in *Populus* and their sequence characteristics (bp, base pair; aa, amino acids; D, Dalton)

**Gene symbol**	**Gene locus**	**Arabidopsis orthologs locus**	**ORF(bp)**	**Exons**	**Protein**			**Number of CCCH motif**
					**Length(aa)**	**Mol. Wt (kD)**	**PI**	
PtC3H1	POPTR_0007s01080.1	AT1G03790.1	1152	1	383	43.28	7.25	2
PtC3H2	POPTR_0017s04510.1	AT1G03790.1	1149	1	382	43.02	8.04	2
PtC3H3	POPTR_0002s21790.1	AT1G04990.1	1353	7	450	48.77	8.06	5
PtC3H4	POPTR_0014s15770.1	AT1G04990.1	1341	7	446	48.23	8.06	5
PtC3H5	POPTR_0009s04610.1	AT1G07360.1	1596	4	531	59.51	7.38	1
PtC3H6	POPTR_0009s04630.1	AT1G07360.1	1596	4	531	59.30	7.54	1
PtC3H7	POPTR_0004s23730.1	AT1G10320.1	780	4	259	31.16	4.73	1
PtC3H8	POPTR_0002s02670.1	AT1G19860.1	1278	4	425	46.16	6.38	1
PtC3H9	POPTR_0005s11800.1	AT1G19860.1	1722	5	573	63.08	8.47	1
PtC3H10	POPTR_0005s25760.1	AT1G19860.1	1263	5	420	45.18	7.05	1
PtC3H11	POPTR_0002s07810.1	AT1G21580.1	6363	10	2120	23.27	8.85	5
PtC3H12	POPTR_0005s20550.1	AT1G21580.1	6228	11	2075	22.86	8.33	5
PtC3H13	POPTR_0001s36810.1	AT1G30460.1	2010	9	669	73.34	6.90	3
PtC3H14	POPTR_0011s09220.1	AT1G30460.2	837	2	278	30.73	8.19	3
PtC3H15	POPTR_0001s02330.1	AT1G32360.1	1134	2	377	40.01	6.97	3
PtC3H16	POPTR_0003s09240.1	AT1G32360.1	1173	2	390	42.10	6.77	3
PtC3H17	POPTR_0004s09410.1	AT1G66810.1	942	1	313	34.79	8.04	2
PtC3H18	POPTR_0005s15100.1	AT1G66810.1	1017	2	338	37.23	8.22	2
PtC3H19	POPTR_0005s19930.1	AT1G66810.1	888	3	295	32.01	9.19	1
PtC3H20	POPTR_0010s12860.1	AT1G68200.2	801	3	266	30.20	8.28	2
PtC3H21	POPTR_0006s08100.1	AT1G75340.1	1299	11	432	46.50	8.66	1
PtC3H22	POPTR_0008s14320.1	AT2G02160.1	2229	3	742	81.56	5.41	3
PtC3H23	POPTR_0010s10850.1	AT2G02160.1	2208	3	735	81.12	5.59	3
PtC3H24	POPTR_0014s16340.1	AT2G05160.1	1731	8	576	65.58	6.87	1
PtC3H25	POPTR_0006s25080.1	AT2G19810.1	1173	1	390	42.62	7.86	2
PtC3H26	POPTR_0006s13510.1	AT2G20280.1	1116	8	371	42.43	5.49	2
PtC3H27	POPTR_0008s04540.1	AT2G20280.1	1092	8	363	41.12	5.06	2
PtC3H28	POPTR_0016s08500.1	AT2G20280.1	1083	8	360	41.15	5.06	2
PtC3H29	POPTR_0308s00200.1	AT2G20280.1	1077	8	358	40.76	5.48	2
PtC3H30	POPTR_0018s02840.1	AT2G24830.1	1536	4	511	56.91	4.83	1
PtC3H31	POPTR_0011s05550.1	AT2G33835.1	2010	5	669	73.52	7.52	1
PtC3H32	POPTR_0008s06940.1	AT2G40140.1	2040	1	679	73.87	7.68	2
PtC3H33	POPTR_0010s19520.1	AT2G40140.1	1839	4	612	66.62	6.77	2
PtC3H34	POPTR_0012s12760.1	AT2G40140.1	2025	1	655	71.58	7.01	2
PtC3H35	POPTR_0001s27370.1	AT2G41900.1	2133	1	710	77.59	6.94	2
PtC3H36	POPTR_0006s05240.1	AT2G41900.1	2208	1	735	79.81	6.40	2
PtC3H37	POPTR_0006s05250.1	AT2G41900.1	2106	1	701	76.47	6.36	2
PtC3H38	POPTR_0009s06580.1	AT2G41900.1	2187	1	728	79.28	6.50	2
PtC3H39	POPTR_0016s05410.1	AT2G41900.1	2199	1	732	79.67	6.50	2
PtC3H40	POPTR_0017s06030.1	AT2G47680.1	3171	13	1056	119.38	6.77	2
PtC3H41	POPTR_0013s08490.1	AT3G02830.1	1560	11	519	57.42	8.42	4
PtC3H42	POPTR_0013s08500.1	AT3G02830.1	318	2	105	11.16	7.34	1
PtC3H43	POPTR_0019s08030.1	AT3G02830.1	1338	7	445	48.48	8.51	5
PtC3H44	POPTR_0008s22730.1	AT3G06410.1	1434	7	477	50.53	8.62	5
PtC3H45	POPTR_0003s06730.1	AT3G08505.1	1548	10	515	59.51	9.26	2
PtC3H46	POPTR_0004s14200.1	AT3G08505.1	1395	6	464	52.58	7.66	4
PtC3H47	POPTR_0009s06730.1	AT3G08505.1	1155	8	384	42.48	8.10	4
PtC3H48	POPTR_0016s04690.1	AT3G12130.1	858	3	285	30.04	9.91	3
PtC3H49	POPTR_0008s07980.1	AT3G12680.1	1425	11	474	51.29	8.27	5
PtC3H50	POPTR_0010s18390.1	AT3G12680.1	1599	12	532	57.88	8.08	6
PtC3H51	POPTR_0005s06240.1	AT3G18640.1	2577	3	858	95.28	7.73	3
PtC3H52	POPTR_0007s03970.1	AT3G18640.1	2583	3	860	95.64	8.69	3
PtC3H53	POPTR_0003s06010.1	AT3G19360.1	795	2	264	28.80	9.99	3
PtC3H54	POPTR_0004s17670.1	AT3G19360.1	1155	2	384	42.89	8.48	3
PtC3H55	POPTR_0009s13310.1	AT3G19360.1	1026	2	341	38.08	7.94	3
PtC3H56	POPTR_0007s02620.1	AT3G21810.1	1314	11	437	49.05	9.09	1
PtC3H57	POPTR_0001s34600.1	AT3G27700.1	2946	5	981	107.01	6.94	1
PtC3H58	POPTR_0017s10830.1	AT3G27700.1	2961	5	986	107.25	7.20	1
PtC3H59	POPTR_0009s04950.1	AT3G47120.1	1053	4	350	41.27	8.97	1
PtC3H60	POPTR_0012s09400.1	AT3G48440.1	1434	6	477	53.20	5.48	5
PtC3H61	POPTR_0015s10050.1	AT3G48440.1	1494	7	497	56.30	5.13	5
PtC3H62	POPTR_0005s11920.1	AT3G51120.1	4575	11	1524	167.73	5.98	1
PtC3H63	POPTR_0004s16880.1	AT3G51120.1	3339	9	1112	121.35	7.18	1
PtC3H64	POPTR_0007s13760.1	AT3G51120.1	4818	10	1605	176.29	5.97	1
PtC3H65	POPTR_0007s13990.1	AT3G51180.1	1674	4	557	61.21	9.16	1
PtC3H66	POPTR_0001s05760.1	AT3G51950.1	2124	8	707	77.04	6.17	1
PtC3H67	POPTR_0001s26250.1	AT3G51950.1	1911	8	636	69.96	6.43	1
PtC3H68	POPTR_0003s20310.1	AT3G51950.1	2118	8	705	77.16	6.15	1
PtC3H69	POPTR_0009s05520.1	AT3G51950.1	2205	8	734	81.19	5.93	1
PtC3H70	POPTR_0012s13800.1	AT4G25440.1	1359	9	452	48.96	7.68	2
PtC3H71	POPTR_0015s13760.1	AT4G25440.1	1365	9	454	49.48	7.49	2
PtC3H72	POPTR_0018s04720.1	AT4G29190.1	1155	1	384	42.18	7.33	2
PtC3H73	POPTR_0009s12840.1	AT4G38890.1	2115	10	704	77.83	6.58	1
PtC3H74	POPTR_0001s05070.1	AT5G06770.1	915	3	304	32.51	9.80	3
PtC3H75	POPTR_0003s21780.1	AT5G06770.1	948	3	315	33.72	9.77	3
PtC3H76	POPTR_0006s20620.1	AT5G06770.1	1008	4	335	35.18	9.89	3
PtC3H77	POPTR_0016s04590.1	AT5G06770.1	855	3	284	29.92	9.79	3
PtC3H78	POPTR_0001s26240.1	AT5G12440.1	2025	7	674	74.81	5.72	1
PtC3H79	POPTR_0001s28300.1	AT5G12440.1	663	1	220	24.13	7.47	1
PtC3H80	POPTR_0007s12500.1	AT5G12440.1	291	1	96	10.76	8.22	1
PtC3H81	POPTR_0001s26560.1	AT5G12850.1	2028	1	675	73.98	6.76	2
PtC3H82	POPTR_0009s05810.1	AT5G12850.1	1887	1	628	69.16	6.72	2
PtC3H83	POPTR_0010s02320.1	AT5G18550.1	1371	7	456	48.77	8.53	5
PtC3H84	POPTR_0002s00480.1	AT5G42820.1	822	1	273	32.47	8.99	2
PtC3H85	POPTR_0004s20030.1	AT5G42820.1	963	5	320	37.42	9.61	2
PtC3H86	POPTR_0005s27940.1	AT5G42820.1	819	1	272	32.24	9.47	2
PtC3H87	POPTR_0006s11790.1	AT5G42820.1	936	4	311	36.25	9.40	2
PtC3H88	POPTR_0006s15670.1	AT5G56900.2	1821	9	606	67.52	6.90	2
PtC3H89	POPTR_0006s15080.1	AT5G56930.1	2787	7	928	101.34	8.46	3
PtC3H90	POPTR_0001s25960.1	AT5G58620.1	2115	1	704	76.38	6.14	2
PtC3H91	POPTR_0009s05150.1	AT5G58620.1	2067	1	703	74.40	5.97	2

Molecular Wt and PI of *Populus* CCCH proteins have been calculated using DNAman software.

### Comparative analysis of the CCCH genes in *Populus*, *Arabidopsis*, and rice

**Figure 1:**
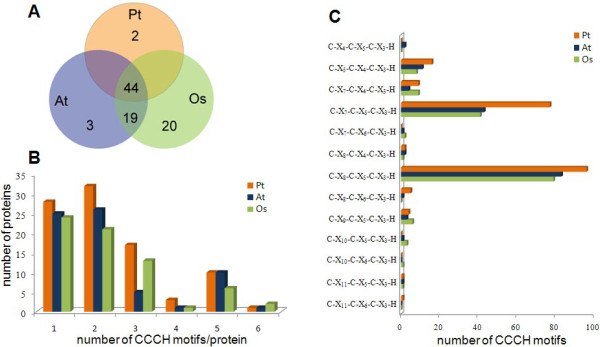
**Statistics on the CCCH proteins from*Populus*(Pt),*Arabidopsis*(At) and rice (Os).** A. Numbers of the CCCH proteins. The number in overlapping zone represents number of homologous genes between three species. B. Numbers of CCCH proteins with 1, 2, 3, 4, 5 or 6 CCCH motifs. C. Numbers of CCCH motifs for each CCCH motif class.

The CCCH family appears to undergone complicated evolution processes and become one of the largest gene families in plants [8]. In the study, we compared the members of CCCH gene family in *Populus* and *Arabidopsis* and rice (Figure 1A) and found that 44 gene clusters were present. Each of the clusters included at least one, up to six counterparts from all of the species we examined, implying the conservation of CCCH genes among *PopulusArabidopsis* and rice. The events that led to the expansion of the 44 CCCH gene clusters in the three species may be very complex, likely involving one or a few round (s) of whole-genome duplication (WGD) followed by a series of tandem duplications and (or) rearrangements during the evolution of certain species. For example, one gene cluster has seven *Populus* CCCH genes (*PtC3H3539, 81* and *82*), but has only two *Arabidopsis* CCCH genes (*AtC3H3056*) and two rice CCCH genes (*OsC3H2450*). This discrepancy suggests that *Populus* CCCH genes may have undergone two rounds of WGDs and one tandem duplication, while the two homologues of either *Arabidopsis* or rice might be created by the segmental duplication (Table 1). Besides those conservative CCCH genes, two, three and twenty CCCH genes were also found unique for *PopulusArabidopsis* and rice, respectively (Figure 1A). These species-specific CCCH genes might be obtained or retained differentially between species during evolution that may lead to different biological functions. Surprisingly, 19 pairs of homologues were identified in both *Arabidopsis* and rice, but not in *Populus*, suggesting that these CCCH genes might not be necessary for wood plant species and therefore have been lost during the evolutionary process.

Previously, it has been suggested that the CCCH gene family contained different numbers and types of CCCH domain in either animals or plants [8,10,33,37]. In this study, we investigated the motif characteristics of the CCCH genes in *PopulusArabidopsis* and rice (Figure 1B). Similar to the other two species, each *Populus* CCCH protein has at least one CCCH motif, and 69.2 % of *Populus* CCCHs have at least two CCCH motifs. As shown in Figure 1C and additional file 2, although the three species had different fractions of CCCH motif types in CCCH gene family, two conventional CCCH motifs, C-X_7_-C-X_5_-C-X_3_-H and C-X_8_-C-X_5_-C-X_3_-H, constituted the largest two groups in all three species, suggesting that the C-X_7–8_-C-X_5_-C-X_3_-H motifs may be an ancestor of other CCCH motifs. Compared to that, 18 % *Populus* CCCH motifs were non-conventional with C-X_5__7__8_-C-X_4_-C-X_3_-H, C-X_8_-C-X_6_-C-X_3_-H, C-X_9__11_-C-X_5_-C-X_3_-H and C-X_11_-C-X_6_-C-X_3_-H. It’s noteworthy that none of *Populus* CCCH proteins contained the C-X_10_-C-X_5_-C-X_3_-H motif that was previously identified to be an abundant non-conventional CCCH motif in *Arabidopsis* and rice [8]. Additionally, a unique C-X_11_-C-X_6_-C-X_3_-H motif was found in *Populus*, suggesting that *PtC3H27* containing this motif may have different binding activity and biological function.

**Figure 2:**
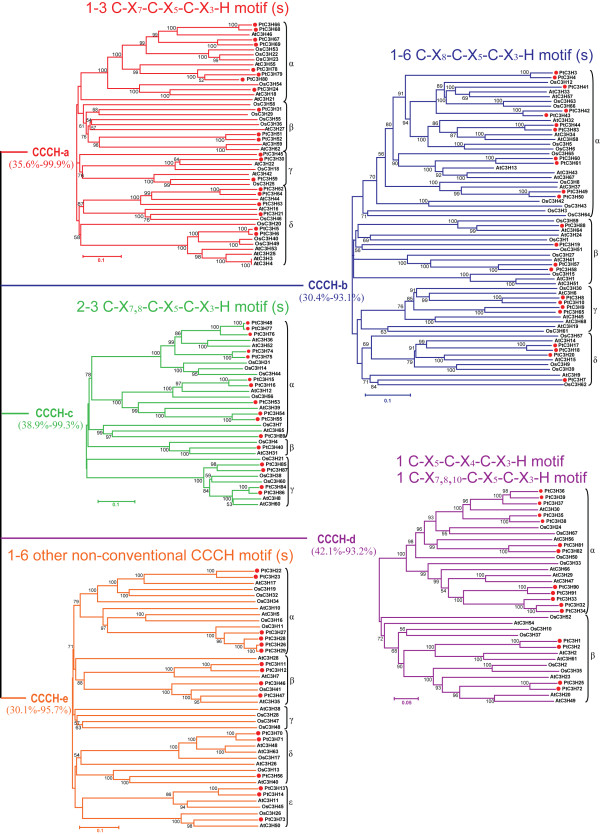
**Phylogenetic trees of full-length CCCH domain proteins from *Populus,Arabidopsis*and rice.** All CCCH proteins of *Populus* (91), *Arabidopsis* (68) and rice (67) were divided into five distinct subfamilies (CCCH-a to CCCH-e) based on the types of CCCH motif. Each protein in subfamily CCCH-a has 1–3 C-X_7_-C-X_5_-C-X_3_-H motif (s), CCCH-b has 1–6 C-X_8_-C-X_5_-C-X_3_-H, CCCH-c has 2–3 C-X_7_-C-X_5_-C-X_3_-H and C-X_8_-C-X_5_-C-X_3_-H, CCCH-d has 1 C-X_5_-C-X_4_-C-X_3_-H and 1 C-X_7,8,10_-C-X_5_-C-X_3_-H, whereas CCCH-e has 1–6 other non-conventional CCCH motifs. The unrooted tree was constructed based on the full-length protein sequences using MEGA 4.0. Numbers at nodes indicate the percentage bootstrap scores and only bootstrap values higher than 50 % from 1,000 replicates are shown. The percentages in the bracket represent protein sequence similarity range for each subfamily, which were obtained using the Smith-Waterman algorithm. *Populus* CCCH proteins were marked with the red dots. The scale bar corresponds to 0.05 or 0.1 estimated amino acid substitutions per site.

To evaluate the evolutionary relationship among the CCCH proteins, a phylogenetic analysis was performed based on the full-length amino acid sequences of *PopulusArabidopsis* and rice. Unfortunately, the obtained phylogenetic tree had low sequence similarity overall, therefore could not exhibit real evolutionary relationship between the different subfamilies (data not shown). These observations might be explained by the divergence of CCCH domains and other non-homologous motifs (e.g. ANK, RRM and KH), especially the diverse CCCH motif types that possess different spacing amino acids between conserved Cys and His residues in each protein. It appears that two conventional CCCH motifs C-X_7, 8_-C-X_5_-C-X_3_-H and one non-conventional C-X_4_-C-X_5_-C-X_3_-H constituted the largest three groups in the CCCH proteins of *PopulusArabidopsis* and rice (Figure 1), additionally, identical CCCH motifs within the same CCCH protein usually have redundant or at least similar functions [35]. Therefore, in this study, based on the types of CCCH motif in each protein, all CCCH proteins of the three species were divided into five subfamilies that were renamed as CCCH-a, b, c, d and e (Figure 2 and Additional file 2) according to the previous method described by Hu and coworkers [34]. Our results demonstrated that five subfamilies has different types of CCCH domain, for example, each protein in subfamily CCCH-a has 1–3 C-X_7_-C-X_5_-C-X_3_-H motif (s), CCCH-b has 1–6 C-X_8_-C-X_5_-C-X_3_-H, CCCH-c has 2–3 C-X_7_-C-X_5_-C-X_3_-H and C-X_8_-C-X_5_-C-X_3_-H, CCCH-d has 1 C-X_5_-C-X_4_-C-X_3_-H and 1 C-X_7,8,10_-C-X_5_-C-X_3_-H, whereas CCCH-e has 1–6 other non-conventional CCCH motifs. For each subfamily, the phylogenetic tree was constructed based on the full-length protein sequences using the Neighbor-Joining (NJ), Minimal Evolution (ME) and Maximum Parsimony (MP) algorithms, respectively. The tree topologies produced by these three algorithms were identical except for the interior branches (data not shown). Therefore, only the NJ phylogenetic tree was subject to further analysis in our study.

The NJ phylogenetic trees indicated that the CCCH genes exhibited an alternating distribution of monocots and eudicots in each subfamily, implying that an ancestral set of CCCH genes already may exist before the monocot-eudicot divergence (Figure 2). Further analysis revealed that the number of *PopulusArabidopsis* and rice CCCH genes varied in most subfamilies, for example, the number of *Populus* CCCH-b, d and e were nearly equalled to that of *Arabidopsis* and rice, while the number of *Populus* CCCH-c genes was the largest among these three species, and was almost two-fold of the other two species. These variation of CCCH-c genes among these three species suggested the subsets of genes with the C-X_8_-C-X_5_-C-X_3_-H motif may have been either lost in *Arabidopsis* and rice or acquired in the *Populus* lineages after divergence from their last common ancestor. The observation of gene duplication in *Populus* was also presented in the analysis of other plant transcription factor families such as NAC [34], bHLH [38], Dof [39], and WRKY [40]. We further examined the subgroups within each CCCH subfamily. Based on the >50 % bootstrap values, each CCCH subfamily can be divided into 3–5 clades designated as clade α, β, γ, δ, and ϵ (Figure 2). It’s noteworthy that clade α in subfamily CCCH-c and CCCH-d was mainly composed of a subset of *Populus* CCCH paralogues. In contrast, clade β in subfamily CCCH-d and clade © in subfamily CCCH-e included more CCCH proteins from *Arabidopsis* and rice than from *Populus*.

### Phylogenetic analyses of the CCCH proteins in *Populus*

**Figure 3:**
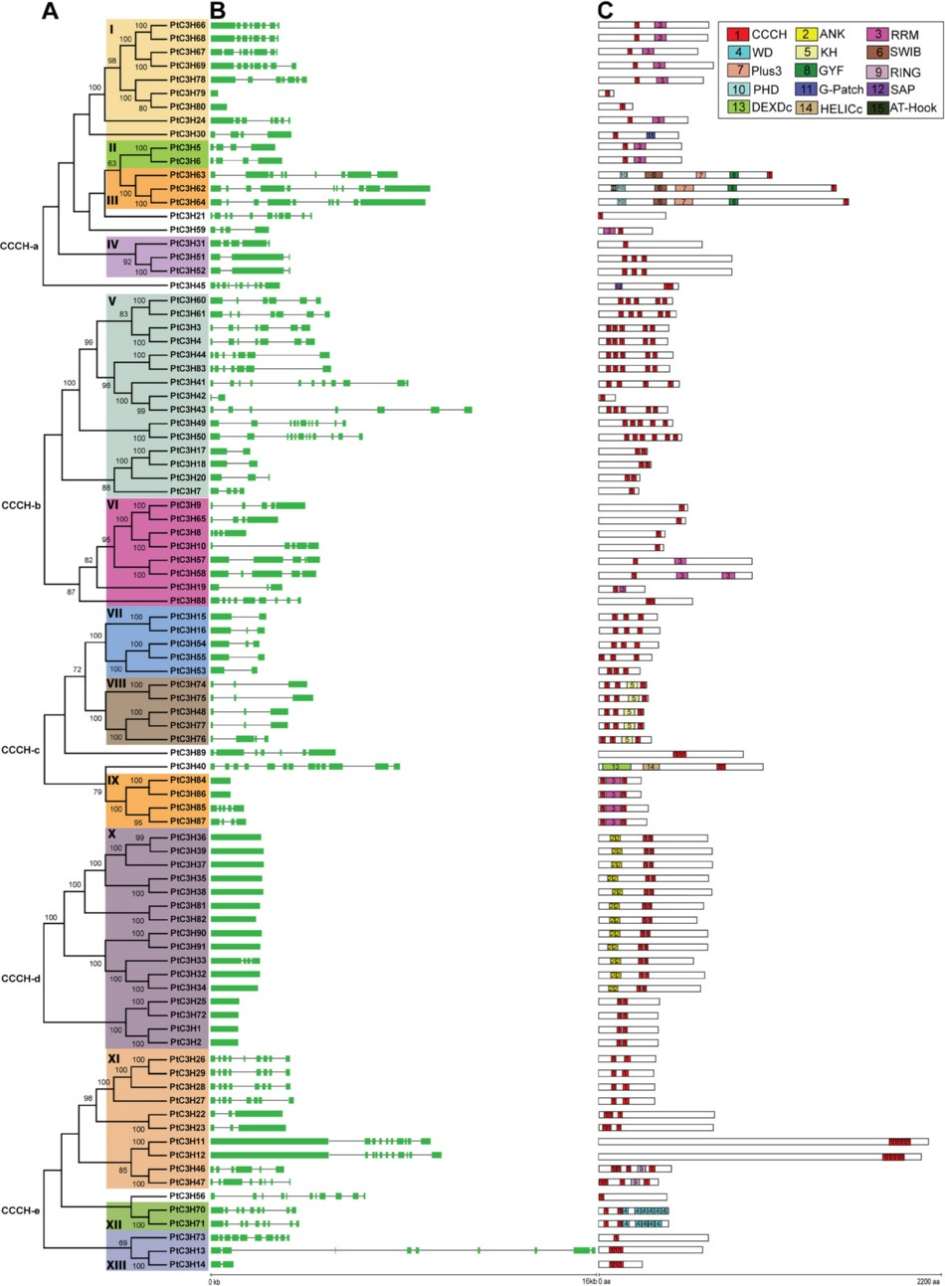
**Phylogenetic relationships, gene structure and motif compositions of *Populus*CCCH genes.** A. Multiple alignments of 91 full-length CCCH proteins from *Populus* were conducted by Clustal X 1.83 and the phylogenetic tree was constructed using MEGA 4.0 by the Neighbor-Joining (NJ) method with 1,000 bootstrap replicates. The percentage bootstrap scores higher than 50 % are indicated on the nodes. The tree shows 13 major phylogenetic subfamilies (subfamily I to XIII marked with different color backgrounds) with high predictive value. B. Exon/intron organization of *Populus* CCCH genes. Green box represents exon and black line represents intron. The sizes of exons and introns can be estimated using the scale at bottom. C. Schematic representation of the conserved motifs in *Populus* CCCH proteins elucidated by SMART online. Each colored box represents a motif in the protein with motif name indicated in box on the right side. The length of the protein and motif can be estimated using the scale at bottom. Refer to Additional file 4 for details of individual motif.

To evaluate the evolutionary relationships between *Populus* CCCH proteins, a phylogenetic analysis of the 91 *Populus* protein sequences was performed (Figure 3A). Similar to the *Arabidopsis* CCCH proteins, the numbers of CCCH motifs in *Populus* CCCH proteins and the spacing amino acids between adjacent CCCH zinc-finger motifs varied. Therefore, the individual phylogeny was constructed using *Populus* full-length CCCH protein sequences based on each subfamily in Figure 2. For statistical reliability, Bootstrap analysis was conducted with 1000 replicates.

The *Populus* CCCH family was further divided into 13 subfamilies (I to XIII) based on the > 50 % bootstrap values (Figure 3A). Within each subfamily, CCCH domains (e.g. C-X_7_-C-X_5_-CX_3_-H in subfamily I and C-X_8_-C-X_5_-CX_3_-H in subfamily V) and other domains (e.g. RRM domain in subfamily I and KH domain in subfamily VIII) are highly conserved, suggesting strong evolutionary relationships among subfamily members. Compared to the eight *Arabidopsis* CCCH subfamilies, the number of *Populus* subfamilies is much larger, implying a genome expansion of *Populus* CCCH counterparts. It is well-known that there are nearly 8000 pairs of paralogous genes in *Populus* genome [28]. Based on the phylogenetic analysis, we identified 34 paralogous pairs from all 91 *Populus* CCCH genes (Table 2), with the percentage (74.7 %) similar to that of *Populus* NAC (60.1 %) [34] and *Populus* GST (69.1 %) gene families [36].

**Table 2:** Divergence between Paralogous CCCH Genes Pairs in *Populus*

**No.**	**Gene 1**	**Gene 2**	**K_s_**	**K_a_**	**K_a_/K_s_**	**Duplication**	**Motif/Gene structure Characteristics**	**Gene Expression**
1	PtC3H1	PtC3H2	0.3237	0.0906	0.2797	O	1	AA
2	PtC3H3	PtC3H4	0.2939	0.0477	0.1624	W	1	AA
3	PtC3H5	PtC3H6	0.0416	0.0088	0.2104	T	1	AA
4	PtC3H8	PtC3H10	0.2961	0.1455	0.4913	W	2	AB
5	PtC3H9	PtC3H65	0.3851	0.1906	0.4950	W	2	No
6	PtC3H11	PtC3H12	0.2979	0.1402	0.4706	W	2	AB
7	PtC3H13	PtC3H14	0.4247	0.1629	0.3835	W	2	No
8	PtC3H15	PtC3H16	0.3063	0.0483	0.1577	W	2	AC
9	PtC3H17	PtC3H18	0.3111	0.0603	0.1939	W	1	AC
10	PtC3H22	PtC3H23	0.2919	0.1136	0.3893	W	1	AB
11	PtC3H25	PtC3H72	0.3609	0.0571	0.1582	O	1	No
12	PtC3H26	PtC3H29	0.0125	0.0078	0.6247	O	2	No
13	PtC3H32	PtC3H34	0.1084	0.0482	0.4446	O	1	No
14	PtC3H35	PtC3H38	0.2903	0.0678	0.2334	W	1	AC
15	PtC3H36	PtC3H39	0.3407	0.0632	0.1855	W	1	No
16	PtC3H42	PtC3H43	0.3935	0.1055	0.2681	W	3	No
17	PtC3H44	PtC3H83	0.2825	0.0712	0.2521	O	1	AC
18	PtC3H46	PtC3H47	1.4585	0.7131	0.4889	O	2	AC
19	PtC3H48	PtC3H77	0.0234	0.0048	0.2064	T	1	No
20	PtC3H49	PtC3H50	0.2805	0.0684	0.2440	O	3	AB
21	PtC3H51	PtC3H52	0.2720	0.0812	0.2985	W	1	No
**22**	PtC3H54	PtC3H55	0.2172	0.0637	0.2930	W	2	No
**23**	PtC3H57	PtC3H58	0.1967	0.1130	0.5745	W	3	AA
**24**	PtC3H60	PtC3H61	0.2951	0.1372	0.4650	W	2	AB
**25**	PtC3H62	PtC3H64	0.2644	0.1031	0.3900	W	3	No
**26**	PtC3H66	PtC3H68	0.2405	0.0373	0.1552	W	1	AC
**27**	PtC3H67	PtC3H69	0.3284	0.1028	0.3131	O	1	No
**28**	PtC3H70	PtC3H71	0.2795	0.0818	0.2925	W	1	AB
**29**	PtC3H74	PtC3H75	0.2405	0.0364	0.1512	W	1	AB
**30**	PtC3H79	PtC3H80	0.2565	0.1364	0.5318	O	1	No
**31**	PtC3H81	PtC3H82	0.2465	0.1066	0.4326	W	1	AC
**32**	PtC3H84	PtC3H86	0.4725	0.0678	0.1435	W	1	AD
**33**	PtC3H85	PtC3H87	2.4099	0.1624	0.0674	O	2	AD
**34**	PtC3H90	PtC3H91	0.2513	0.0496	0.1972	W	1	AB

Gene pairs were identified at the terminal nodes (>80 % identical) of the phylogenetic tree shown in Figure 1. Synonymous (Ks) and nonsynonymous substitution (Ka) rates are presented for each pair. Gene pairs created by tandem duplication (T), whole genome duplication (W), or other (O) events are indicated in the table. Motif/Gene structure characteristics of gene pairs were divided into three groups: 1, identical exon/intron structure and motif composition; 2, identical motif and variable gene structure; 3, less conserved exon/intron structure and motif composition. Gene expression patterns based on microarray data (GSE13990) are categorized into four classes: AA, both of duplicates were expressed in non-overlapping tissues; AB, both duplicates had the same expression patterns; AC, expression tissues of one duplicate completely covered the other; AD, expression tissues of both duplicates were overlapping but different; No, no data for one duplicate is present in the microarray.

### Gene structure and conserved motifs of *Populus* CCCH genes

To gain further insights into the structural diversity of CCCH genes, we compared the exon/intron organization in the coding sequences of individual CCCH genes in *Populus* (Figure 3B). Most closely related members in the same subfamilies share similar exon/intron structures either in terms of intron numbers or exon length, which was consistent with the characteristics defined in the above phylogenetic analysis. For instance, the CCCH genes in subfamily VII and VIII contained one to three introns while those in subfamily X possessed no introns with exception of *PtC3H33*. In contrast, although the intron phase is remarkably conserved within *Populus* CCCH V subfamily (Additional file 3), the gene structures of subfamily V appeared to be more variable in terms of intron numbers, which may be indicative of exon shuffling during the evolution [41].

To discover conserved motifs shared among related proteins within the family, we used both MEME (Multiple Expectation Maximization for Motif Elicitation) [42] and SMART online server (http://smart.embl-heidelberg.de/) to predict the putative motifs. Surprisingly, most motifs cannot be observed except for five motifs when using the MEME program with the previous reported parameters [8,34]. In contrast, 15 distinct motifs were identified in *Populus* CCCH proteins by SMART (Figure 3C and Additional file 4), which is similar to those of *Arabidopsis* CCCH proteins [8]. As expected, most of the closely related members had common motif compositions, suggesting functional similarities among the CCCH proteins within the same subfamily. It is noteworthy that subfamily X, the largest subfamily containing 16 members, had been divided into two subgroups. In addition to two CCCH motifs (C-X_7_-C-X_5_-CX_3_-H and C-X_5_-C-X_4_-C-X_3_-H), each protein of subgroup I consists of two ankyrin (ANK) repeat motifs which were shown to play a variety of roles in diverse molecular processes such as transcriptional initiation, ion transportation and signal transduction [43,44]. The proteins in subfamily VIII mostly contained well-defined RNA-binding domain KH, suggesting their potential role involved in RNA binding [45]. These specific motifs of the subfamily members may, by some extent, attribute to the functional divergence of CCCH genes [8].

Gene structure and conserved motifs of 34 CCCH paralogous pairs in *Populus* were further investigated (Figure 3B, C and Table 2). Three categories were significantly classified based on two counterparts’ gene structure and motif composition of each gene pairs. Among them, 20 gene pairs possessed the identical exon/intron structure and motif composition, 9 pairs exhibited the identical motif and variable gene structure in term of intron number and length, and 5 pairs shared relatively less conserved exon/intron structure and motif composition (Table 2). Moreover, the difference of gene organization and motif composition between the paralogous pairs suggested that they may be functionally divergent.

### Chromosomal location and gene duplication

**Figure 4:**
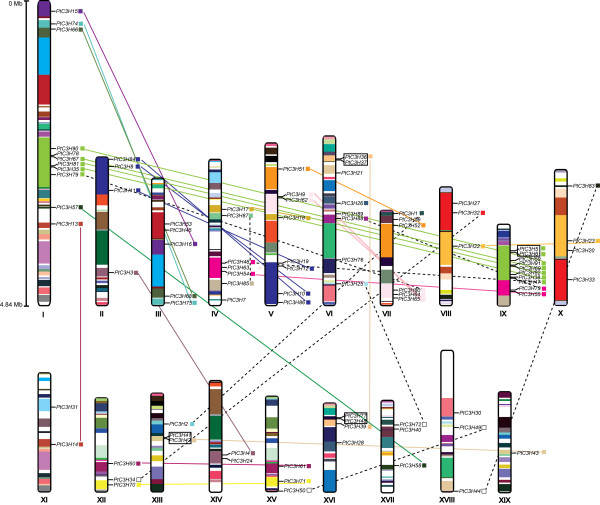
**Chromosomal locations of *Populus*CCCH genes.** 90 CCCH genes are mapped to the 19 Linkage Groups (LG), while only one gene (*PtC3H29*) resides on unassembled scaffolds. The schematic diagram of *Populus* genome-wide chromosome organization arisen from the salicoid genome duplication event was adapted from Tuskan *et al.*, (2006) [30]. Segmental duplicated homologous blocks are indicated with the same color. Small boxes connected by colored line (two types) indicate corresponding sister gene pairs, of which the genes connected by solid line locate in the duplicated blocks, while one or both of genes connected by the dashed line was (were) not observed in the duplicated blocks. Tandemly duplicated genes are encompassed in the black boxes. Scale represents the length (4.84 Mb) of chromosome I.

90 of the 91 *Populus* CCCH genes were physically located on 19 Linkage Groups (LG) of *Populus*, while only one gene (*PtC3H29*) was remained on as-of-yet unattributed scaffold fragments (Figure 4). The distribution of *Populus* CCCH genes among the chromosomes appeared to be uneven: LG XI, XIV and XIX harbour one or two CCCH genes, while relatively high densities of CCCHs were discovered in some locations on LG I, IV, V, VI, and IX. Particularly, CCCHs located on the duplicated fragments of LG I and IX are arranged in clusters.

Previous analysis of the *Populus* genome indicated that the paralogues within gene family were mainly derived from the whole-genome duplication event in the Salicaceae (salicoid duplication) occurred 60 to 65 million years ago, with occasional tandem duplication and transposition events such as retroposition and replicative transposition [46]. To determine the evolutionary relationship between *Populus* CCCH genes, the distribution of CCCHs were further investigated within the 163 recently identified duplicated blocks [30]. Of the 90 mapped CCCHs, only nine were located outside of the duplicated blocks, while 90 % (81of 90) were located in duplicated regions. Furthermore, 16 block pairs covered 24 CCCH paralogous pairs by whole genome duplication, and 23 block pairs only harboured CCCHs on one of the blocks and lack the corresponding duplicates, suggesting that dynamic changes may have occurred following segmental duplication which results in the loss of some genes.

Four adjacent CCCH gene pairs were found within a distance less than 9 kb on the duplication blocks, which may result from tandem duplication in either the inverse or same orientation (Figure 4). Similar results were also reported in the analysis of other *Populus* gene families [34,36,47]. Alignment analysis of protein sequences using the Smith-Waterman algorithm (http://www.ebi.ac.uk/Tools/psa/) showed that four pairs (PtC3H5/6, PtC3H36/37, PtC3H41/42 and PtC3H48/77) had high sequence similarities (≧80 %) between two counterparts of each gene pair and therefore meet the standards as tandem duplicates. Analysis of CCCH paralogous pairs showed that 22 out of 34 gene pairs remained in conserved positions on segmental duplicated blocks, suggesting that these genes may result from genome duplication (Figure 4 and Table 2). Our study further indicated that the retention rate of duplicated genes was relatively high (44/91, 48.4 %) that was consistent with the recent reports of other gene families in *Populus*[34,47]. Among the non-genome duplicated gene pairs, three genes were located on duplicated segments while their counterparts not on any duplicated blocks, two counterparts of the three paralogous pairs were located separately on divergent rather than homologous duplicated blocks, one gene pair (*PtC3H49*/*50*) were not on any duplicated blocks, and one gene (*PtC3H26*) was located on segmental duplicate blocks with its counterpart (*PtC3H29*) not mapped to LGs yet (Figure 4 and Table 2). Together, the diverse duplication events contributed to the complexity of CCCH gene family in the *Populus* genome.

The ratio of nonsynonymous versus synonymous substitutions (Ka/Ks) is an indicator of the history of selection acting on a gene or gene region [48]. Ratios significantly <0.5 suggest purifying selection for both duplicates [49]. A summary of Ka/Ks for 34 CCCH paralogous pairs is shown in Table 2. The result suggested that all gene pairs had evolved mainly under the influence of purifying selection except for three pairs (*PtC3H26/29PtC3H57/58* and *PtC3H79/80*).

Based on the genomic organization of CCCH genes, we could conclude that segmental duplications contributed significantly to the evolution of CCCH gene family and redundancy resulting from duplication is common in *Populus* genome, which were also observed in other *Populus* gene families [36,39,50,51]. It is reported that approximately 33.4 % of predicted genes originated from salicoid genome-wide duplication and 15.6 % from tandem duplication on a genome scale analysis in *Populus*[30]*.* Our studies indicates that *Populus* CCCH gene family possesses higher segmental duplication ratio (62.9 %) and lower tandem duplication ratio (11.8 %), which are dramatically different from the average. This high retention rate of segmental duplication and low retention rate of tandem duplication are also in consistency with the previous studies on other gene families [34,36,47,51].

### Nucleon-cytoplasm shuttling and RNA-binding proteins

All *Arabidopsis* CCCH proteins have previously been predicted to locate in nucleus by the SubLoc v1.0 software and the subsequent experimental verifications of several CCCH genes such as *AtHUA* and *AtSZF1*[8,20]. However, recently progress suggests that 79.4 % *Arabidopsis* CCCH genes may be nucleocytoplasmic shuttle proteins due to the presence of Leucine-rich Nuclear Export Signal (NES) that seems to be essential for the trafficking of CCCH proteins from the nucleus to cytoplasm [8]. Furthermore, Pomeranz *et al*. experimentally confirmed that *Arabidopsis* Tandem Zinc Finger (TZF) family including 11 CCCH genes can indeed shuttle between the nucleus and cytoplasm, all of which contained the NES sequences [52,53]. To predict the sub-cellular localization of *Populus* CCCH genes, 91 full-length protein sequences were used separately as input sequences in the program WoLF PSORT (http:// wolfpsort.org/). Not surprisingly, all *Populus* CCCH members, similar to that of *Arabidopsis* orthologues [8], were predicted to localize in nucleus (data no shown). To further examine whether 91 *Populus* CCCH proteins have NES sequences or not, a program using widely accepted NES consensus [LV]-x (2, 3)-[LIVFM]-x (2, 3)-L-x-[LIMTKD] was written according to previous study [8]. Of the 91 proteins, 62 (68.1 %) have putative NES sequences (Additional file 5), suggesting that most *Populus* CCCH proteins might be nucleocytoplasmic shuttle proteins involved in signal transduction events [54].

**Figure 5:**
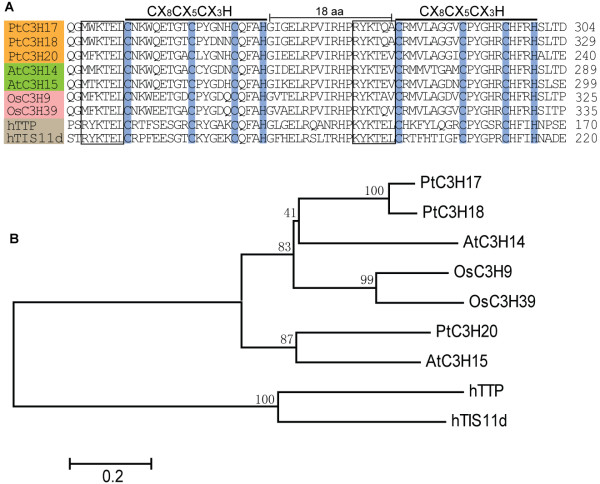
**Sequence alignments of the putative RNA binding proteins.** Amino acid sequence analysis of the typical tandem zinc finger (TZF) domain in nine proteins (PtC3H17, PtC3H18, PtC3H20, AtC3H14, AtC3H15, OsC3H9, OsC3H39, hTTP and hTIS11d). B. Phylogenetic tree of the nine representative TZF RNA binding proteins. The unrooted tree was inferred by MEGA 4.0 with neighbor-joining method after the alignment of the full-length amino acid sequences of the nine genes selected. The number beside the branches represents bootstrap value based on 1,000 replications. The scale bar corresponds to 0.2 estimated amino acid substitutions per site.

Among these nucleocytoplasmic shuttle proteins mentioned above, PtC3H17, PtC3H18 and PtC3H20 all contain two identical C-X_8_-C-X_5_-C-X_3_-H motifs separated by 18 amino acids (Figure 5A), and therefore were regarded as the typical TZF family proteins [52,55]. It is well known that TZF proteins can bind to class II ARE element in 3'-UTR of target mRNAs to promote their deadenylation and degradation [53,56]. Therefore, we speculated that the three *Populus* TZF proteins might as well have RNA-binding abilities. Further comparison analysis revealed that besides TZF motifs, PtC3H17, *Pt*C3H18 and *Pt*C3H20 also shared the conservative lead-in sequence at the N-termini (MW/F/M/TKTEL or R/KYKTE/A/QV/A) that may provide the critical parts of the RNA-binding surface (Figure 5A). Phylogenetic analysis indicated that PtC3H17, *Pt*C3H18 and *Pt*C3H20 were the closest homologs to their *Arabidopsis* counterparts AtC3H14 and AtC3H15, suggesting that this type of proteins is more evolutionary conservative within eudicots than others (Figure 5B). It has recently been shown that AtTZF1 (AtC3H23, At2g25900) was induced by wounding and MeJA stress [52]. Therefore we investigated digital expression of the three *Populus TZF* genes based on the microarray data (GSE16786) and found that both wounding and MeJA can significantly stimulate the expression of *PtC3H17* and *PtC3H18* (data no shown)*.* However, no microarray data was available for *PtC3H20*.

### Expression patterns of *Populus* CCCH genes in various tissues

**Figure 6:**
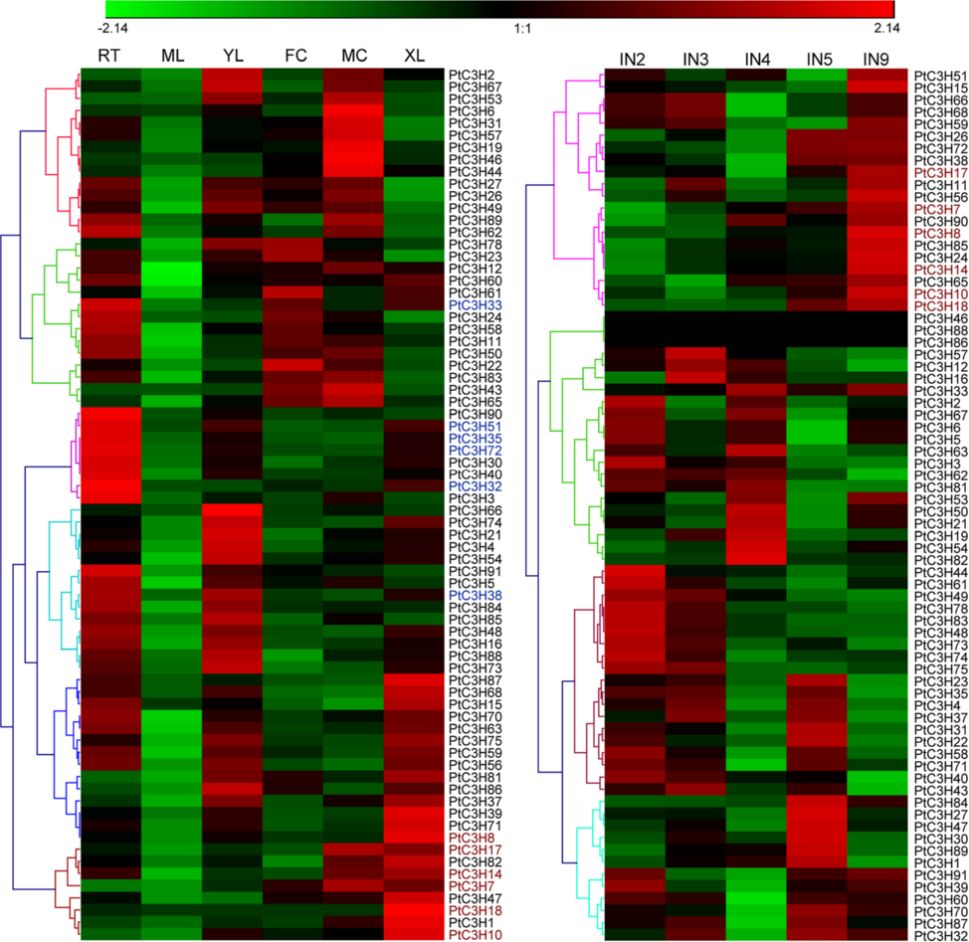
**Hierarchical clustering of expression profiles of *Populus*CCCH genes in different tissues.** The expression data were gene-wise normalized and hierarchical clustered based on Pearson correlation. Color scale at the top of each dendrogram represents log2 expression values. The genes highlighted in color (blue and red) were selected for validation by RT-qPCR. Rt, roots; ML, mature leaves; YL, young leaves; FC, female catkins; MC, male catkins; XL, differentiating xylems. IN2, IN3, IN4, IN5 and IN9 represent stem internode 2 to 9 (plastochron index) of an actively growing young tree under field conditions. Among them, the IN3 stem contains well developed primary vascular tissues. IN9 contains well developed secondary xylems.

Whole genome microarray has been proved to be a useful means of studying gene expression profiles in *Populus*[34,51]. To gain insight into the expression patterns of *Populus* CCCH genes in various tissues, a comprehensive analysis was conducted based on *Populus* microarray data generated by Wilkins and Dharmawardhana [50,57]. Because 19 CCCH genes do not have the corresponding probe sets in the microarray dataset, we only analysed the expression profiles of the remaining 72 CCCH genes (Figure 6 and Additional file 5). Most *Populus* CCCHs genes demonstrate distinct tissue-specific expression patterns except for mature leaves, where all have low transcriptional levels (Figure 6A). Of the *Populus* 72 CCCH genes we examined, 34 showed the highest transcript accumulations in roots, 24 in young leaves, 12 in female catkins, 21 in male catkins and 22 in differentiating xylems. These distinct expression patterns were significantly different from that of *Arabidopsis* or rice CCCH genes where the majority of CCCH genes were expressed in all tissues (roots, inflorescences, leaves and seeds) as illustrated by MPSS and EST data [8]. Although it is generally thought that orthologous genes from different species may retain similar temporal and spatial expression patterns [58,59], the discrepancy of gene expression between *Populus* and *Arabidopsis* might be arisen from either the data origin of Microarray experiments or the evolutionary consequences that more *Populus* CCCH homologs are needed in *Populus* development.

We further examined the gene expression patterns of the *Populus* CCCH paralogous genes. Of the 34 CCCH gene pairs, 13 genes (*PtC3H9, 132529343642, 52556469, 79* and 80) do not have corresponding probe sets on Affymetrix microarray. Therefore, only the remaining 21 paralogous pairs were analyzed. As illustrated in Table 2 and Figure 6, these CCCH genes displayed four distinct expression patterns. In the first category which covered four gene pairs, two gene duplicates were expressed in non-overlapping tissues, suggesting different functions. In the second category, both duplicates of all eight gene pairs shared almost identical expression patterns with respect to the tissues examined. The third category covered seven pairs of duplicate genes. The tissues where one duplicate highly expressed belong to part of its paralogous duplicate. The fourth category only contained two gene pairs (*PtC3H84*/*86* and *PtC3H85*/*87*), which were all homologues of *Arabidopsis AtC3H60* (*At5g42820*). The expression patterns of the two counterparts in each gene pair were partially overlapping but different. It is noteworthy that most gene pairs created by the whole-genome duplication event mostly fell within the second and third categories, with both of the duplicates showing a similar expression pattern. In contrast, one gene pair (*PtC3H5*/*6*) created by tandem duplication belongs to the first category and had different expression pattern. The four categories of expression patterns of paralogs indicate that CCCH gene pairs have diverged quickly after segmental or tandem duplication. It is generally thought that the duplicated genes may undergo divergent fates during subsequent evolution such as nonfunctionalization (loss of original functions), neofunctionalization (acquisition of novel functions), or subfunctionalization (partition of original functions), which may be indicated by divergence in their expression patterns [60,61]. We speculate that the *Populus* CCCH gene pairs with distinct expression patterns from the first category might have undergone neofunctionalization, whereas gene pairs with overlapping expression patterns from the third or fourth category suggest subfunctionalization during subsequent evolution.

**Figure 7:**
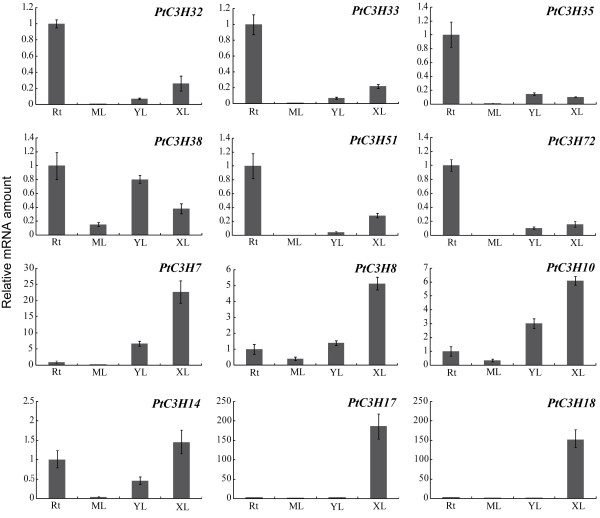
**Expression analysis of selected *Populus* CCCH genes using quantitative real-time RT-PCR (RT-qPCR).** The relative mRNA abundance of 12 *Populus* CCCH genes was normalized with respect to reference gene UBQ10 in different tissues. The bars represent standard deviations (SD) from three technical repeats. RT, roots; ML, mature leaves (4 ~ 6 internodes from top); YL, young leaves (1 ~ 3 internodes); XL, differentiating xylems.

Identification of the genes predominantly expressed in xylems provides an important clue for their functions during the development of secondary cell walls in *Populus*[62,63]. To identify such CCCH genes, another heatmap was generated based on the microarray data (GSE13043) [57], in addition to the above results. As showed in Figure 6B, most of the CCCHs exhibited different expression levels in *Populus* stem segments (IN2, IN3, IN4, IN5, and IN9). IN2 and IN3 represent the vascular tissue of primary growth, mainly including primary xylem and primary phloem. IN5 and IN9 have well developed secondary phloem tissues and secondary xylem vessels, as well as fibres with well lignified secondary walls [57]. Expression of these CCCH genes suggested they may play the special roles during each phase of cell wall biosynthesis. Expression patterns of most *Populus* CCCH genes in IN9 (Figure 6B) were basically identical to the patterns in xylems (Figure 6A). We selected six genes (*PtC3H78101417*, and *18*) that are highly expressed in xylem as well as in IN9 to further verify the validation of previous Microarray data using RT-qPCR. All six genes tested demonstrated the highest expression level in xylem compared to other tissues we examined, which was in good agreement with the microarray profiles (Figure 7). Of these six genes, two *Populus* CCCH genes (*PtC3H17* and *PtC3H 18*) exhibited particularly high transcript accumulations in xylem. *AtC3H14*, the *Arabidopsis* orthologues of *PtC3H17* and *PtC3H18* genes, was previously shown to play key role in the regulatory network of secondary cell wall biosynthesis [24,25]. Taken together, this study may provide a further solid basis to select xylem-specific genes for related functional validation.

### Expression profiling of *Populus* CCCH genes under drought stress

**Figure 8:**
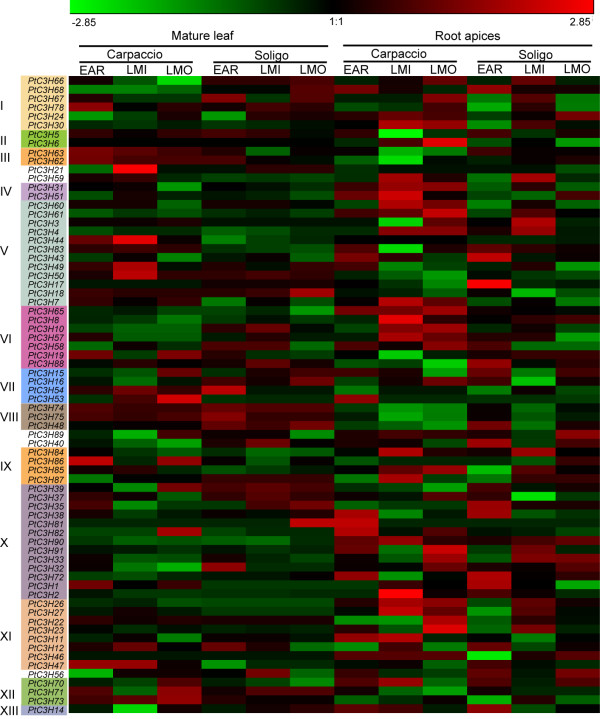
**Expression profile of *Populus* CCCH genes under drought condition.** Color bar at the top represents log2 expression values. The corresponding genes in 13 subfamilies are shown on the left side. Stage of drought treatment, organs and genotypes are showed on the top. Genotype ‘Carpaccio’ productivity is less hampered by drought than that of ‘Soligo’. EAR, a short-term water deficit by withholding irrigation 36 hours; LMI, 10-day-long response to mild stress; LMO, 10 day-long response to moderate stress.

A subset of *Arabidopsis* CCCH genes have previously been shown to play crucial roles in drought stress-response. In order to better understand the roles of *Populus* CCCH genes in drought tolerance, we reanalysed the expression profiles of all *Populus* CCCH genes in response to drought stresses using the publicly available Microarray data. As illustrated in Figure 8, the expression of drought-treated trees was obtained from different organs (root apices and mature leaves) and genotypes (*Populus* Soligo and Carpaccio) [64]. Consistent with the transcriptional changes to most drought-driven transcription factors in *Populus* roots and leaves [64], most *Populus* CCCH genes, especially CCCH IV, VI, X and XI, showed more significant response in root apices than mature leaves when subject to drought stresses (Figure 8). A possible explanation is that compared to leaves, roots sense the edaphic water deficits to send chemical signals to shoots and to further maintain the root growth despite reduced water availability can contribute to drought tolerance through water foraging [65]. It also appears that the *Populus* CCCH genes are differentially regulated in response to various drought stresses between two different *Populus* phenotypes. Under prolonged drought stress (LMI, long-term response to mild stress; and LMO, long-term response to moderate stress), the expression of drought-driven *Populus* CCCH genes in root apices displayed less significant changes in water deficit-sensitive genotype ‘Soligo’ than that in less sensitive genotype ‘Carpaccio’. Under early drought response (EAR), contrary to the responses to prolonged drought stress, most of the CCCH genes in Soligo roots exhibited more drought-driven regulation than that of Carpaccio. Interestingly, a subset of CCCH genes mainly distributed in subfamily V and XVII were up-regulated in leaves under all drought conditions. The diverse drought-mediated responses suggested that the up- or down-regulated *Populus* CCCH genes might fall into different physiologically relevant patterns in root or leaf system according to iterative group analysis (iGA) [64,66].

**Figure 9:**
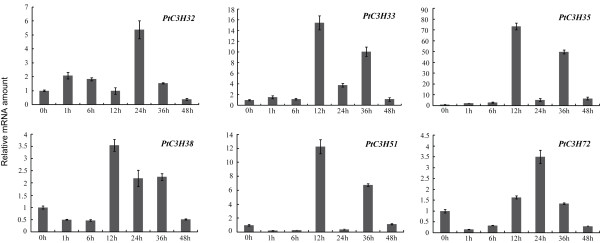
**RT-PCR analysis of 6 selected *Populus* CCCH genes under drought condition. **X-axis represents time points after drought treatment. Y-axis represents relative gene expression values normalized to reference gene UBQ10. Bars are standard deviations (SD) from three technical repeats.

To screen *Populus* CCCH genes regulated by drought stress, RT-qPCR was used to validate six candidate genes (*PtC3H3233353851* and *72*) that are highly induced by drought stresses in roots in Microarray data. The results showed that consistent with the Microarray data, the six genes not only exhibited the root-specific expression patterns and were but also regulated by drought stresses in roots (Figure 7 and Figure 8). Further analysis revealed that the six selected genes displayed different expression patterns between the two genotypes (Figure 8). This result was partially similar to the *Arabidopsis* orthologues, which showed that drought stress had significant effect on expression of most genes by RT-PCR analysis [8]. For the diverse expression patterns of CCCH genes under drought stress, a plausible explanation is that poplar is sensitive to water deprivation, as well as drought tolerance varies considerably between genotypes [67-70]. To examine the detailed gene expression changes of *Populus* CCCH genes under drought stresses, RT-qPCR analysis was performed on the six *Populus* CCCH genes using 4-month-old *P. deltoides* seedlings (see the materials). The drought-driven gene expression patterns of the six *Populus* CCCH genes can be divided into two groups based on the time point of their transcript abundances reaching the maximum (Figure 9). One group (*PtC3H32* and *PtC3H72*) accumulated the highest transcripts at 24 hrs after drought treatment, whereas transcription level of other group (*PtC3H3335, 38, 51*) exhibited two peaks at 12 hrs and 36 hrs after the drought treatment. Moreover, the expression patterns were not identical between the members within each subgroup. Further analysis found that *Arabidopsis* homologs (*AtC3H29*/*30*/*38*/*49*) of the six genes tested have also been identified to be involved in drought response [8]. We speculated that the diverse expression patterns of the CCCH genes suggested that they might be involved in different drought signal network. It would be, therefore, interesting to undertake further functional studies of these CCCH genes at mRNA metabolism level to establish the interactions of biochemical pathways that are activated during drought stress response.

Recently, the accumulating evidences show that *Populus* water-deficit transcriptome is not only influenced by the genotype, but also by the time of day [69,70]. In the hybrid poplar DN34 genotype, a large number of drought-induced genes were significantly induced in midday, compared to dawn and late in the day [69]. By contrast, the time point of treatment has less significant effect on drought-driven genes transcript in the pure *P. balsmifera* genotypes compared with the hybrid poplar genotypes [70]. In the current study, we attempt to collect the samples in the afternoon and dawn to reduce the impact of diurnal rhythm on drought-induced genes transcript, despite the pure *P. deltoides* genotype also used as the materials.

It is noteworthy that the real-time PCR results were in good agreement with the microarray data sets in the study, although the species (*P. deltoides*) used for qPCR were different from the ones (*P. balsamifera*, *P. trichocarpa* and *P. x canadensis*) producing microarray data (see the materials). The reasons underlying the similar expression patterns may be high conservation of the genes tested between the four species. Observation of their proteins found that they possessed identical motif compositions, with 1–2 CCCH or (and) ANK domains. Furthermore, their similar expression patterns between GSE13990 and GSE13043 also suggested the conversed functions within the four species. Further data need to be experimentally confirmed.

## Conclusions

Characteristics of CCCH gene family is preliminarily documented in model plant *Arabidopsis* and rice. However, this family has not been studied in the model tree *Populus* to date. In the present study, a comprehensive analysis including phylogeny, chromosomal location, gene structure, conserved motifs, and expression profiling of CCCH gene family in *Populus* was performed. A total of 91 full-length CCCH genes in *Populus* genome were identified, of which most contain more than one CCCH motif and a non-conventional C-X_11_-C-X_6_-C-X_3_-H motif unique for *Populus* was found. *Populus* CCCH genes were clustered into 13 distinct subfamilies based on phylogenetic analysis. In each subfamily, the characteristics of exon/intron structure and motif compositions were relatively conserved. A high proportion of CCCH genes were found to distribute preferentially at the duplicated blocks, suggesting that segmental duplications contribute significantly to the expansion of *Populus* CCCH gene family. Comparative analysis showed that 34 gene pairs were created by different duplication types, which displayed four categories of digital expression pattern in six tissues across different developmental stages, suggesting some categories have undergone subfunctionalization during evolutionary process. Furthermore, a subset of *Populus* CCCH genes was identified to be possibly involved in wood formation and drought response. In addition, 62 CCCH genes were found to contain NES sequences and might be nucleocytoplasmic shuttle proteins. Among them, three had the typical characteristics of TZF proteins. The new information obtained could help in the selection of appropriate candidate genes for further functional characterization.

## Methods

### Identification and nonmenclature of genes

*Populus trichocarpa* genome database (release 2.1, http://www.phytozome.net/poplar.php) was searched to identify CCCH motif-containing-proteins using Basic Local Alignment Search Tool algorithms BLASTP and TBLASTN with the published plant and animal CCCH proteins as query sequence and with E-value cutoff set as 1e-005. All obtained protein sequences were examined for the presence of the CCCH motif by the Hidden Markov Model of Pfam (http://pfam.sanger.ac.uk/search) [71] /SMART (http://smart.embl-heidelberg.de/) [72] tools. The sequences recognized by Pfam (PF00642) /SMART (Sm00356) were thus considered to be *Populus* CCCH proteins. Manual reannotation was then performed to correct the predicted genes using online web server FGENESH (http://linux1.softberry.com/berry.phtml) [73]. The sequences filtered by the stringent chosen conditions were further examined for the CCCH domain using InterProScan program (http://www.ebi.ac.uk/Tools/InterProScan/) [74]. Finally, the obtained genes were compared with the CCCH family in PlnTFDB (http://plntfdb.bio.uni-potsdam.de/v3.0/) [75] and DPTF (http://dptf.cbi.pku.edu.cn/) [76]. The 91 CCCH genes from *Populus* have been named from *PtC3H1* to *PtC3H91* according to previously available nomenclature.

### Phylogenetic analysis

Multiple alignments of amino acid sequences were performed by ClustalX (version 1.83) program and were manually corrected. The phylogenetic trees were generated with MEGA 4.0 [77] using the Neighbor-Joining (NJ), Minimal Evolution (ME) and Maximum Parsimony (MP) methods [78]. Bootstrap analysis with 1,000 replicates was used to evaluate the significance of the nodes. Pairwise gap deletion mode was used to ensure that the divergent domains could contribute to the topology of the NJ tree. Gene clusters refer to the homologs within three species (*PopulusArabidopsis* and rice) were identified based on NCBI web (http://www.ncbi.nlm.nih.gov/).

### Sequence properties and chromosomal location

The amino acid sequences of the CCCH proteins were analyzed for physicochemical parameters by DNAman software (Lynnon Biosoft Co., Canada), and subcellular localization was predicted by WoLF PSORT program (http:// wolfpsort.org/) [79]. The exon/intron organization of CCCH genes was generated online with Gene structure display server (GSDS) (http://gsds.cbi.pku.edu.cn/) [80]. Structural motif annotation was performed using the SMART program mentioned above. Identification of homologous chromosome segments resulting from whole-genome duplication events was accomplished as described previously [30]. Blocks with the same color represent homologous chromosome segments. Tandem gene duplications were identified according to criteria described elsewhere [81]. Genes separated by five or fewer gene loci in a range of 100 kb distance were considered to be tandem duplicates. Synonymous (Ks) and nonsynonymous substitution (Ka) rates were calculated according to previous study [82].

### Microarray analysis

The genome-wide microarray data were obtained from the Gene Expression Omnibus database at the National Center for Biotechnology Information under the series accession numbers GSE13990 (from *P. balsamifera*), GSE13043 (from *P. trichocarpa*), GSE17223 (from *P. x canadensis*), and GSE17230 (from *P. x canadensis*). Probe sets corresponding to the putative *Populus* CCCHs were identified using an online Probe Match tool available at the NetAffx Analysis Center (http://www.affymetrix.com/). For genes with more than one probe sets, the median of expression values was considered. When several genes have the same probe set, they are considered to have same transcriptional profile. The expression data were gene-wise normalized and hierarchical clustered based on Pearson coefficients with average linkage in the Genesis (version 1.75) program [83].

### Plant material collection

Young leaf (internodes 1 ~ 3 from top), mature leaf (from internodes 4 ~ 6), developing xylem (from the basal internodes) and root tissues of one-year-old *P. deltoides* plants grown in the greenhouse (16 h light/8 h dark, 25 °C ~ 28 °C) were harvested respectively. Drought stress treatment was conducted following the previous method with minor modification [84]. Briefly, the 4-months-old *P. deltoides* seedlings were removed from the pots and exposed on filter paper to air with 70 % RH at 25 °C under dim light. Roots were collected at different time points (0 h, 1 h, 6 h, 12 h, 24 h, 36 h, and 48 h) after treatment, respectively. To reduce the impact of diurnal rhythm on drought-induced gene transcript, samples were collected from 17:00 (0 h). Three replicates from three independent plants were collected per harvest and were immediately frozen in liquid nitrogen and stored at −80 °C until required.

### Real-time RT-PCR verification

Total RNAs were isolated with the RNeasy mini kit (Qiagen, USA) according to the manufacturer’s instructions. The RNA preparation was then treated with Dnase I and first strand synthesis of cDNA was performed by using oligo (dT) primer and M-MLV RT (Promega). Primers were designed using Beacon Designer v7.0 (Premier Biosoft International, USA) with melting temperatures 58 ~ 60 °C, primer lengths 20 ~ 24 bp and amplicon lengths 90 ~ 150 bp. Each primer was checked using BLAST tool of NCBI database with filter off for its specificity for respective gene, which was further confirmed by melting curve analysis from realtime PCR reaction. Details of primers are given in additional file 6.

Real-time RT-PCR was conducted on LightCycler® 480 Detection System (Roche, Germany) using SYBR Premix Ex Taq (TaKaRa, Japan) according to the manufacturer’s instructions. To normalize the variance among samples, UBQ10 was used as internal reference gene. Baseline and threshold cycles (Ct) were determined with 2^nd^ maximum derivative method using the LightCycler ® 480 Software release 1.5.0. Relative gene expression with respect to UBQ10 was determined as described previously [85].

## Authors’ contributions

GC carried out all the analysis and interpreted the results. RH and DZ participated in the data mining. QG, RZ, YC and PC helped in *Populus* materials collection and total RNA extraction. GZ and YK conceived the project, supervised the analysis and critically revised the manuscript. All authors read and approved the final manuscript.

## Supplementary Material

Additional file 1 ** A complete list of 91 CCCH gene sequences identified in the present study.** Genomic DNA sequences are obtained from Phytozome (http://www.phytozome.net /poplar, release 2.1). Amino acid sequences are deduced from the corresponding coding sequences. Click here for file

Additional file 2 **The conserved motifs and related data of *Populus,Arabidopsis*and rice CCCH proteins.** Conserved motifs were identified in *Populus* CCCH proteins using the two programs of MEME and SMART. The characteristics of *Populus*, *Arabidopsis* and rice CCCH motifs were summarized as well*. * Click here for file

Additional file 3 **Exon/intron organization of the CCCH genes in *Populus.*** Exons and introns are represented by green boxes and black lines, respectively. The number indicates the splicing phases of the CCCH genes. 0, phase 0; 1, phase 1; 2, phase 2. Click here for file

Additional file 4 **The putative NES sequences in *Populus *CCCH proteins.** These sequences were detected by regular expression consensus [LV]-x (2, 3)-[LIVFM]-x (2, 3)-L-x- [LIMTKD] according to the previous method [8]. Click here for file

Additional file 5 **The probes and microarray data of *Populus*CCCH genes.** Microarray data corresponds to expression analysis showed in Figure 6 and Figure 8. Click here for file

Additional file 6 **Primer sequences of 12 selected *Populus* CCCH genes for RT-qPCR analysis.** The sequence, length, Tm, GC% and product sizes of primers used for RT-qPCR. Click here for file
