# Supplementary material for: Comprehensive analysis of CCCH zinc finger family in poplar (Populus trichocarpa)
Source: BMC Genomics. 2012 Jun 18;13:253. doi: 10.1186/1471-2164-13-253 (PMC3427045; doi:10.1186/1471-2164-13-253)
Supplement: Additional file 3 — Exon/intron organization of the CCCH genes in Populus. Exons and introns are represented by green boxes and black lines, respectively. The number indicates the splicing phases of the CCCH genes. 0, phase 0; 1, phase 1; 2, phase 2. [file 1471-2164-13-253-S3.doc]

**
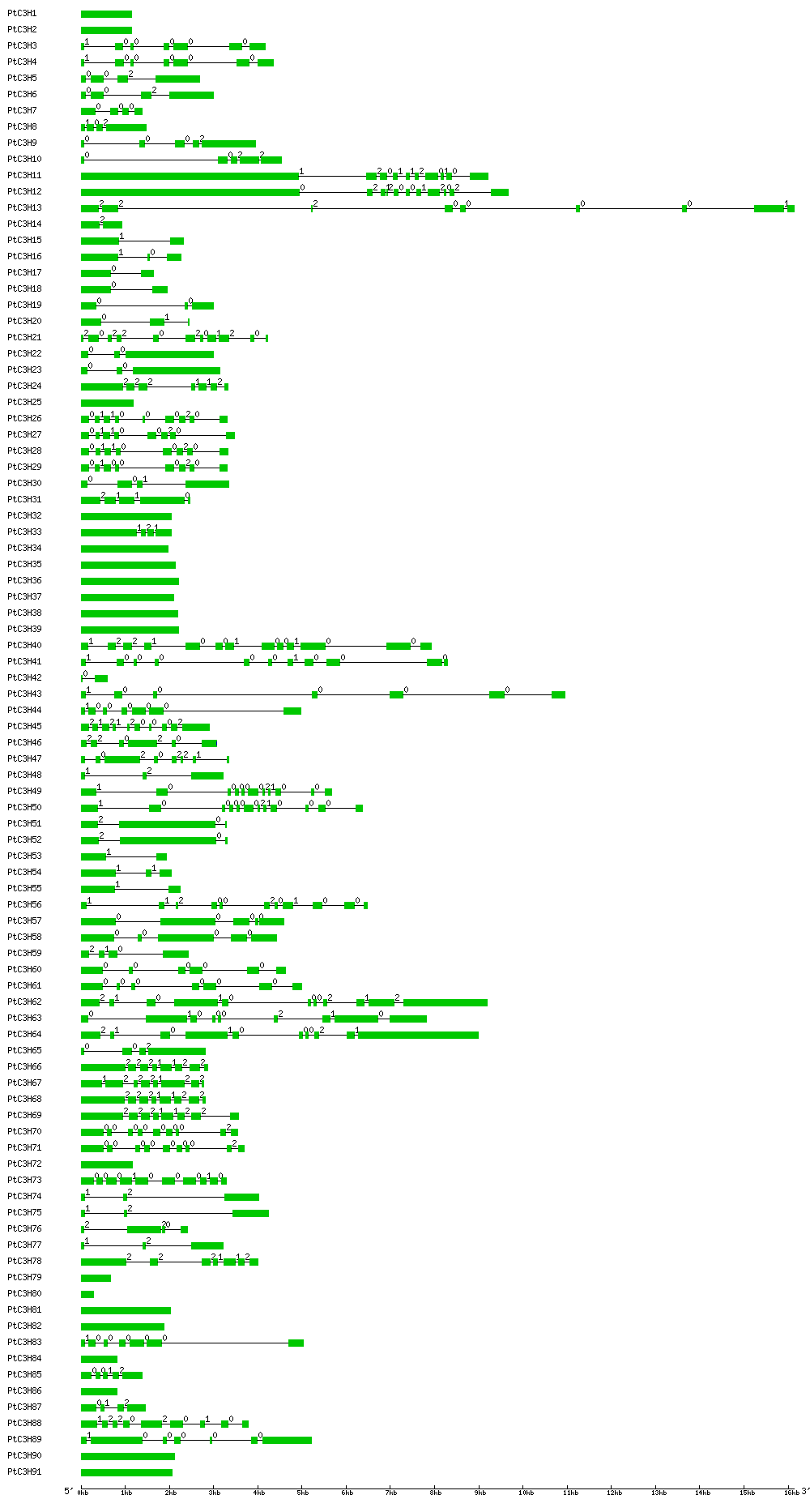
**

**Additional file 2:** Exon/intron organization of the CCCH genes in *Populus*. Exons and introns are represented by green boxes and black lines, respectively. The number indicates the splicing phases of the CCCH genes. 0, phase 0; 1, phase 1; 2, phase 2.
